# Supplementary material for: GraphCompass: spatial metrics for differential analyses of cell organization across conditions
Source: Bioinformatics. 2024 Jun 28;40(Suppl 1):i548–57. doi: 10.1093/bioinformatics/btae242 (PMC11256915; doi:10.1093/bioinformatics/btae242)
Supplement: btae242_Supplementary_Data [file btae242_supplementary_data.zip › btae242_Supplementary_Data/Ali_and_Kuijs.173.sup.1.pdf]

A. Supplementary Methods

A.1. Diffusion Method

Diffusion, in the context of graphs, refers to the process of spreading a certain amount of an imaginary substance (like information, heat, etc.) across the nodes of a graph over time. Diffusion on graphs can be intuitively understood through the analogy of balls connected by springs. When you impart energy to one ball in the system (for example, by hitting or pushing it), this energy is represented by the ball's movement. As the ball starts moving, it stretches or compresses the springs connected to it (the edges in the graph). This, in turn, transfers energy to the balls (nodes) at the other ends of these springs. Balls directly connected to the moving ball receive the energy first, and then the energy propagates to others in a ripple-like effect. The overall structure of the graph (how balls are connected by springs) affects the energy diffusion pathway and rate. Diffusion on graphs is implemented by NetLSD [Tsitsulin et al., 2018], a Python library that encodes a so-called "trace signature" to capture the energy diffusion process. The trace signature is computed as follows: Given graph G, calculate its normalized Laplacian as

$$\mathcal{L} = I - D^{-\frac{1}{2}} A D^{-\frac{1}{2}}. \tag{7}$$

A and D are the adjacency and degree matrix of G, respectively. Next, we compute the closed-form solution to the heat equation associated with the normalized Laplacian, which is defined as

$$\frac{\partial u_t}{\partial t} = -\mathcal{L} u_t, \tag{8}$$

where u_t represents the imaginary "energy" of a given node at time t. The solution to the heat equation is then computed as

$$H_t = e^{-t\mathcal{L}} = \sum_{j=1}^{n} e^{-t\lambda_j} \phi_j \phi_j^T. \tag{9}$$

Here, $H_{ij,t}$ quantifies the amount of energy transferred from node v_i to node v_j at time t. λ_j and ϕ_j represent the jth eigenvalue and eigenvector, respectively, of the Laplacian with Dirichlet boundary conditions. As a last step, we compute the heat trace h_t as the trace of H_t, such that

$$h_t = tr(H_t) = \sum_j e^{-t\lambda_j}. \tag{10}$$

To compare two graphs, we simply compute the L_2 distance between the corresponding heat traces computed at different times t,

$$d(G, G') = \sqrt{\sum_{t=1}^{n} (h_t - h'_t)^2}. \tag{11}$$

Alternatively, we can cluster the heat traces to reveal sample-level similarities. The clusters can be visualized using, for example, a UMAP. Samples that cluster together are similar in terms of spatial organization.
