# Supplementary material for: GraphCompass: spatial metrics for differential analyses of cell organization across conditions
Source: Bioinformatics. 2024 Jun 28;40(Suppl 1):i548–57. doi: 10.1093/bioinformatics/btae242 (PMC11256915; doi:10.1093/bioinformatics/btae242)
Supplement: btae242_Supplementary_Data [file btae242_supplementary_data.zip › btae242_Supplementary_Data/Ali_and_Kuijs.173.sup.2.pdf]

Table 1. Comprehensive overview of methods for differential analysis of cellular organization in spatial omics data across conditions.

Method	Cell-type Abundance	Cell-type Organization	Neighborhood/ Niche Analysis	Tissue-level Architecture	Code Availability	Comments
**Cluster-based**						
CellCharter [Varrone et al., 2023]			X		X	Requires hyperparameter tuning
MENDER [Yuan, 2024]			X		X	Requires hyperparameter tuning
SOTIP [Yuan et al., 2022]			X	X	X	Unflexible predefined neighborhood shapes and sizes; potential for false negatives
**Image-based**						
Qualitative image analysis [Risom et al., 2022]	X		X			Dataset-specific analysis; not generalizable or reproducible
**Simple statistics**						
Qualitative niche analysis [Kuppe et al., 2022]	X		X		X	Dataset-specific analysis; not generalizable
Cell-type composition analysis [Wei et al., 2022]	X				X	Dataset-specific analysis; not generalizable
**Graph-based**						
Graph neural networks (GNN) [Wu et al., 2022, Hu et al., 2024]	X		X	X	X	Computationally expensive; requires GPU and a large sample size
**GraphCompass**	X	X	X	X	X	Resource efficient, optional hyperparameter tuning, no need for GPU; comprehensive documentation and tutorials; insightful visualization functions
